# Towards data-driven electricity management: multi-region uniform data and knowledge graph

**DOI:** 10.1038/s41597-024-04310-z

**Published:** 2025-01-09

**Authors:** Vid Hanžel, Blaž Bertalanič, Carolina Fortuna

**Affiliations:** Jozef Stefan Institute, Ljubljana, 1000 Slovenia

**Keywords:** Energy management, Research data

## Abstract

Due to growing population and technological advances, global electricity consumption is increasing. Although CO_2_ emissions are projected to plateau or slightly decrease by 2025 due to the adoption of clean energy sources, they are still not decreasing enough to mitigate climate change. The residential sector makes up 25% of global electricity consumption and has potential to improve efficiency and reduce CO_2_ footprint without sacrificing comfort. However, a lack of uniform consumption data at the household level spanning multiple regions hinders large-scale studies and robust multi-region model development. This paper introduces a multi-region dataset compiled from publicly available sources and presented in a uniform format. This data enables machine learning tasks such as disaggregation, demand forecasting, appliance ON/OFF classification, etc. Furthermore, we develop an RDF knowledge graph that characterizes the electricity consumption of the households and contextualizes it with household-related properties enabling semantic queries and interoperability with other open knowledge bases like Wikidata and DBpedia. This structured data can be utilized to inform various stakeholders towards data-driven policy and business development.

## Background & Summary

Increased energy consumption that comes with population growth and technological advances raises environmental concerns, therefore many public and private institutions are committing to emission cuts and increased energy efficiency solutions. For instance, the European Union has committed to improving energy efficiency by at least 32.5% by 2030 compared to the 2020 levels (https://ec.europa.eu/clima/eu-action/climate-strategies-targets/2030-climate-energy-framework_en). Efforts in meeting such targets are underway and involve (i) increasing the efficiency of existing technologies, such as engines, home appliances, and electricity grids; (ii) moving towards renewable sources and (iii) developing incentives for various stakeholders. While success stories are being reported, such as the case of Denmark where in 2017, 70.6% of the total electricity production was generated by renewable energies, including wind (67.4%), biomass (25.9%), solar (3.4%), biogas (3.1%), and hydro (0.1%)^1^, half of the world’s electricity is still generated from natural gas and coal^2^. Nevertheless, to fulfill projections such as having renewables contribute to 38% of global electricity generation^2^ by 2027, suitable policies and regulations to incentivize all stakeholders towards the desired goal have to be developed. Focusing on achieving the overall sustainability goals, we identify three groups of stakeholders, namely *Governments and regulatory bodies*, *Distribution system operators* and *Household residents* and analyze their role in achieving the targets and the challenges they are confronted with.

### Government and regulatory bodies

**Fig. 1:**
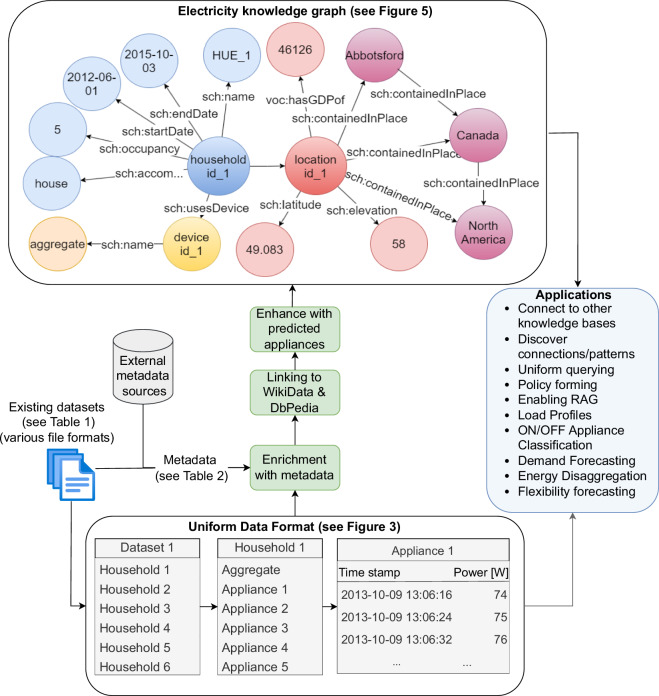
The relationship between the data in the uniform format, knowledge graph and supported applications. The upper box provides a view into the knowledge graph. While the lower box presents the uniform data format. Some of the steps to generating the knowledge graph are showcased in the green boxes, while the blue box showcases the possible applications of our data.

The role of such stakeholders is to design good policies and regulations to achieve an overall increase in the share of renewables in household consumption. In this endeavor, social justice aspects also have to be considered as it has been reported that the capacity of energy users to shift their energy-using practices in time or space, also referred to as flexibility capital, makes some householders more capable of being flexible, than others^3^. Given this, more comprehensive data-driven studies to understand different residential electricity consumption behaviors^4^, effects of moving or rescheduling flexible load in view of peak shaving/clipping, etc. are required as most of the existing studies are limited by the measured metrics, sample size, period, and geographical scale^1,5,6^. For instance, the energy poverty index is typically calculated by the share of disposable income being spent on energy^7^. However, Cong^6^ showed that, when more information is available, namely the outside temperature and exact timings when a household turns on the heating, more accurate metrics can be developed better informing subsidizing policies for such households. Furthermore, in China the government aims to implement a carbon peak initiative, reaching peak carbon emissions by 2030 and achieving carbon neutrality by 2060. The realization of this goal is hindered by the lack of comprehensive CO_2_ emission data^5^. Motivated by such data scarcity issues, we are contributing a multi-region electricity knowledge graph (KG)^8^ and a uniform data format as depicted in Fig. 1 and explained in detail in at the top of the Methods section.

### Distribution system operators

These are companies that aim to deliver electricity with the highest possible efficiency by managing the electric grid effectively. Based on policies, regulations, and market constraints, they are undergoing deep technological and business model transformations. Their distribution grid is being upgraded with electrical and Information and communications technologies (ICT) components becoming a so-called smart grid that allows bidirectional transmission of power and data, creating an automated power grid^9^. With smart grids, every actor, including individual households equipped with solar cells and batteries, pulls and consumes electricity when needed, but can also push and sell energy when it has a surplus, thus becoming a prosumer.

This objective can be supported by accurate and dependable forecasting of energy-related data, which is crucial for optimizing decision-making processes at the grid level. Currently, the difference in peak and low demand is significant, resulting in grid inefficiencies. To combat this peak shaving is required^10^. Demand-side management (DSM) in peak shaving applications is the reduction of energy consumption during peak demand periods^11^. One approach to DSM is Demand response (DR), which encourages end users to shift their use to off-peak hours by increasing tariffs during high demand allowing for a more efficient system utilizing more renewable usage and in turn reducing CO_2_ emissions^12^. The development of such solutions is one of the possible use cases of the proposed Knowledge graph (KG) and uniform data format as is depicted in the blue box in Fig. 1.

### Households

The primary goal of households is to reduce their electricity as much as possible without sacrificing comfort. Occupant behavior significantly influences energy consumption so two households with very similar appliances can have very different energy profiles^13^. Research has shown that providing the consumer with individual appliance electricity usage can induce behavior change that can lead to up to 15% efficiency improvement^14,15^. Providing such data to the consumer is difficult as sub-metering individual appliances is expensive, one solution to this problem is energy disaggregation, which is the task of inferring individual appliance consumption from the aggregate power signal^16^. However, due to a lack of a comprehensive multi-region dataset, it is difficult to develop models that could be deployed across different countries and cultures.

**Fig. 2:**
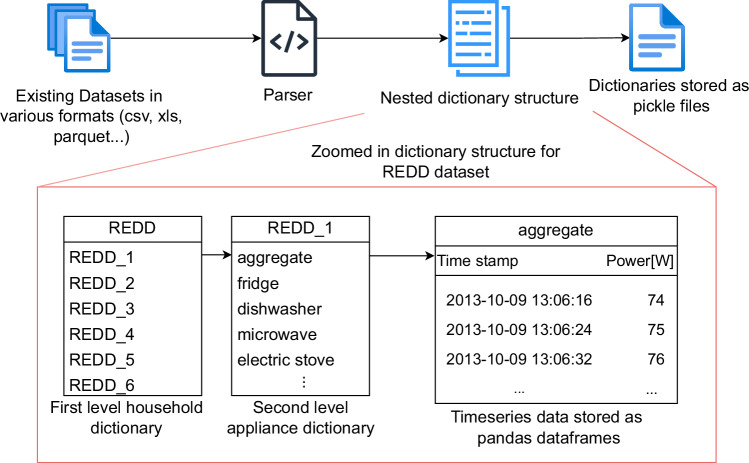
Conversion of raw data into a uniform format. The figure zooms in on the first household from the REDD^30^ dataset, showcasing the nested dictionary structure. Raw datasets, originally in various formats (CSV, parquet, xls), are parsed, cleaned, and organized into a consistent dictionary format, stored as pickle files. This uniform structure simplifies further analysis and integration with other datasets.

To summarize, it can be seen that applications such as electricity flexibility forecasting, peak shaving, demand forecasting, disaggregation, etc. empower the three classes of stakeholders to design better policies and incentive mechanisms in the case of government and regulatory bodies, better and more agile technologies empowering innovative business models in the case of DSOs as well as improved end-user awareness through specific data-driven individualized communication in the case of household consumers. As we focus on domestic electricity consumption, the household is naturally the fundamental unit for data collection, representation, and analysis, and the mentioned applications leverage per household or various types of aggregations of measured data. While there are several per household openly available datasets, their diversity in terms of measured parameters, sampling rate, and duration requires investing extensive effort in pre-processing, therefore preventing larger-scale multi-regional data studies for more robust application development. For instance, existing disaggregation models are developed on max 5 datasets with 29 appliances^17^, and demand forecasting models are usually limited to a single region^18^. To mitigate these shortcomings, we provide 1) a multi-region uniform dataset to serve developing more generic ML models as illustrated in the lower half of Fig. 1 and 2) a structured and enriched form of this data embodied as a knowledge graph, as illustrated in the upper half of Fig. 1, that can be semantically queried and is interoperable with other open structured knowledge bases such as Wikidata and DBpedia. To contribute to better household energy management strategies, this paper proposes the creation of uniform, structured household electricity consumption data compiled from multiple regions globally. Our main contributions are:

- We provide a multi-region household electricity consumption dataset from various parts of the world compiled from existing publicly available datasets in a uniform format enabling data analysis and Machine learning (ML) tasks such as energy disaggregation, load demand forecasting, and appliance ON/OFF classification.
- We propose a comprehensive multi-region electricity KG of residential households across the world. The KG contains detailed household and appliance metadata (data about data) and is linked with external KGs such as WikiData and DBpedia allowing for extensive analysis and semantic querying.
- We provide a model training workflow with a pre-trained ensemble of InceptionTime models adapted for appliance ON/OFF multi-label classification. The workflow encompasses 64 appliances from 10 open-source datasets across different regions of the world, making it the most comprehensive to date.

To further elaborate, the uniform data format contains electricity consumption data that has been standardized into a consistent format. This includes raw aggregate household and individual appliance consumption data, which can be used for various analyses and machine learning tasks, such as time series analysis, anomaly detection, and consumption forecasting. The format of this data can be seen at the bottom of Fig. 1 and a concrete example from the REDD dataset can be seen in Fig. 2.

**Fig. 3:**
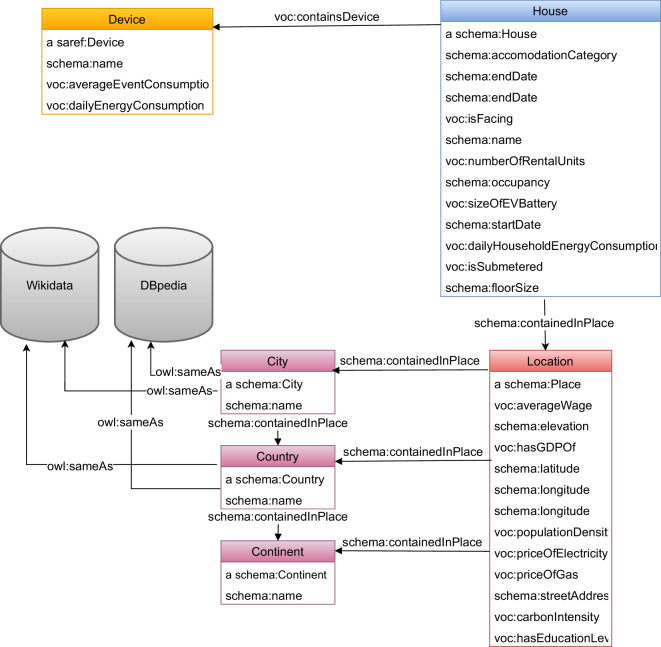
The ontology used for the proposed knowledge graph visualized as a UML class diagram. The tables represent the classes, while the rows in the tables and the arrows between the tables represent the predicates.

On the other hand, the KG doesn’t contain raw electricity consumption data, it only contains high level knowledge such as load profiles and average daily consumption. The KG also contains metadata in structured format extracted from the raw datasets. It also builds upon this metadata by incorporating location based demographic data from external sources as highlighted in Table 2, Fig. 3 and the “Metadata Integration” subsection. The structured nature of the data stored in the knowledge graph allows discovering new connections, such as identifying regions with higher CO_2_ footprints or the correlation between income levels and energy usage. This structured format makes it easier to systematically uncover and utilize these connections, for example guiding policymakers in designing targeted interventions, such as subsidies for low-income areas, to effectively reduce carbon emissions and support equitable transitions to low-carbon solutions. Table 4 summarizes the different data formats and the respective content within.

## Related Work

As discussed in the introduction, disaggregation and load forecasting are the enabling data-driven techniques for reducing CO_2_ footprint. To date, existing studies for such techniques employ a limited number of datasets 2–5 usually located on the same continent^17,19–21^ and a limited number of appliances. To our knowledge, the current highest number of datasets used is 5 with 29 appliances^17^. A more robust evaluation of these techniques necessitates the utilization of a larger dataset encompassing a broader spectrum of appliances and geographically dispersed locations. Furthermore, only a few studies are entirely replicable by also providing simulation scripts, code, or the developed model itself^22,23^. For this purpose, we provide a multi-region household consumption dataset comprising of 20 datasets in a uniform format along with a data preprocessing pipeline and model training workflow enabling further research and development in this field. Unlike traditional tools like NILMTK^24^ which focus on evaluating the accuracy of NILM algorithms, our approach emphasizes knowledge graph generation and the integration of external metadata, enabling analyses for broader insights into energy consumption patterns.

In the domain of energy data and smart home applications, the use of KGs has become increasingly prominent. Several key research efforts have significantly contributed to this field. For instance, the study in^25^ introduces an innovative method for developing smart home applications by leveraging a runtime KG approach, significantly reducing coding requirements by about 85%. This model conceptualizes smart home scenarios and abstracts smart device manageability into runtime KGs, including an automatic generation mechanism for smart home applications to minimize manual coding.

Another notable contribution is from^26^, which presents a method for enhancing ML forecasters in smart homes through the integration of heterogeneous Internet of things (IoT) and Smart energy data into a KG. This research focuses on transforming IoT data into Resource Description Framework (RDF) format suitable for KGs and investigates the impact of this transformation on prediction algorithms. The methodology maps IoT data to ontologies and utilizes federated learning, thus enhancing learning capabilities while maintaining data privacy.

Furthermore^27^, proposes a novel graph mining-based methodology for analyzing and visualizing building operational data, thereby enhancing building energy management. The authors develop a technique to transform operational data into graphs, analyzed using frequent subgraph mining to identify operational patterns. This approach integrates multi-relational and hierarchical building data, offering valuable insights for energy management.

These studies collectively underscore the versatility of KGs in improving energy management and smart home systems. They offer diverse perspectives and methodologies, highlighting the breadth of innovation in this field. Our proposed electricity KG addresses a key limitation in existing research by focusing on residential consumption across multiple global regions. The integration of detailed household, location metadata, and appliance-level data enables a level of granular household analysis currently unsupported by other publicly available datasets.

## Methods

A KG^8^, as depicted at the top of Fig. 1, is a network of interconnected data nodes, where each node represents an entity (e.g. a place, or thing), and the connections (or “edges”) represent relationships between the nodes. Unlike traditional tabular data, which organizes information in rows and columns, a KG allows for a more flexible and integrated view of the data. This structure makes it easier to discover patterns and connections that might be hidden in tables and supports more complex queries, like finding indirect relationships between entities. In the case of an electricity KG, the KG captures metadata and high-level features extracted from raw consumption data, such as average daily household energy usage, appliance information, and location details. For example, it can link a household to its appliances and geographic location, allowing for analysis of how factors like energy prices and population density impact consumption. This interconnected structure offers a more comprehensive understanding of electricity usage compared to traditional data formats, enabling more insightful analysis and decision-making. To develop the proposed KG we employ a standard development process consisting of the following main steps: identifying data, constructing the KG ontology, extracting knowledge, processing knowledge, constructing the KG, and maintaining the KG^28^. This section details the methods used to implement the respective steps and also explains the process of generating the uniform data format.

### Existing open source datasets

To develop the KG, we first identify openly available datasets that are suitable for the electricity characterization of households and are of sufficient quality. Table 1 summarizes the available open-source datasets that are suitable for the proposed KG. The first column of the table identifies the dataset by name, From the second column, it can be seen that the sampling rate of electricity measurements ranges from every second (1 s) in most datasets to every hour (1 h) in the HUE dataset. Other datasets have intermediate rates like 6 s (UKDALE), 8 s (REFIT), and 7 s (HES). The sampling rate is particularly important for ML applications where datasets typically require a consistent sampling rate that can be achieved through upsampling and downsampling. Additionally, tasks such as disaggregation are not possible with sampling rates of 1 min and above. From the third column, it can be seen that the duration of the measurements varies from one month (HEART) to 4.3 years (UK-DALE). Other datasets cover periods from several months to a few years. The length of the datasets is crucial for analyzing occupant behavior, as a sufficiently large sample size is necessary to conduct meaningful analysis. The fourth column tells us about the number of households present in the dataset this information is important as for example from one household in the DRED dataset it’s difficult to draw conclusions about the other households in the area, but if we have a representative sample of the area, for example, 110 953 households in ECDUY dataset, we can do city or even country level electricity consumption analysis. The number of unique appliances from the fifth column is important because it informs on how complete the dataset is. For example ENERTALK dataset has only 7 unique appliances which means that very likely not all appliances in the household are sub-metered. The sixth column of the dataset provides information on the countries and continents where the households are located, predominantly in Europe and North America, with three datasets representing Asia. Geographical location is crucial in understanding differences in electricity consumption, as factors such as climate and cultural practices significantly impact electricity usage patterns. The last column tells us if the dataset contains appliance sub-meter data or only the aggregate power consumption data. This information is important so we know which datasets to pick for different ML tasks.

**Table 1:** Summary of the appliance monitoring datasets used in this work, organized by continent and ordered according to the availability of sub-metered appliance data.

Dataset	Sampl. rate	Time	Households	Appliances	Location	Sub-metered
UKDALE^42^	6 s	4.3 y	5	53	United Kingdom, Europe	Yes
ECO^38^	1 s	8 m	6	21	Switzerland, Europe	Yes
REFIT^31^	8 s	2 y	20	23	United Kingdom, Europe	Yes
DRED^37^	1 s	6 m	1	11	Netherlands, Europe	Yes
SUST2^48^	2 s	96 d	1	18	Portugal, Europe	Yes
HEART^39^	1 s	1 m	4	13	Greece, Europe	Yes
DEKN^49^*	1 min	2.5 y	5	/	Germany, Europe	Yes
DEDDIAG^36^	1 s	3.5 y	15	14	Germany, Europe	Only household 8
IDEAL^50^	7 s	2 y	255	/	United Kingdom, Europe	Some households
UCIML^51^	1 min	4 y	1	/	France, Europe	No
LERTA^52^	6 s	1.5 y	4	/	Poland, Europe	No
SUST1^53^	1 min	3.1 y	50	/	Portugal, Europe	No
REDD^30^	1 s	>1 m	6	17	Massachusetts, USA, North America	Yes
HES**	7 s	5 m	1	39	Canada, North America	Yes
EEUD^54^	1 min	1 y	23	7	Canada, North America	Yes
HUE^46^*	1 h	3 y	22	/	Canada, North America	No
ECDUY^55^*	15 min	1.8 y	110953	/	Uruguay, South America	Some households
IAWE^41^	1 s	73 d	1	9	India, Asia	Yes
ENERTALK^29^	1 s	4 m	22	7	South Korea, Asia	Yes
PRECON^56^	1 min	1 y	42	/	Pakistan, Asia	No

The table provides key details for each dataset, including the sampling rate, the total duration of data collection (Time), the number of households and appliances monitored(if available), and their geographic location. The datasets marked with * are measured in kWh and the rest are in watts. The HES dataset is available at its GitHub repository (https://github.com/ETSSmartRes/HES-Dataset).

### Knowledge Graph and Uniform Data Format Development Methodology

**Fig. 4:**
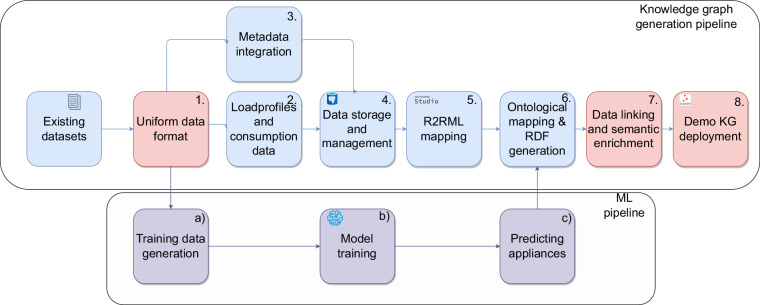
Knowledge graph development methodology. Boxes highlighted in red showcase the steps which are our contributions, the purple boxes highlight the ML pipeline, and the blue boxes represent the KG generation steps.

The proposed KG and uniform data format development process is illustrated in Fig. 4 and consists of a KG generation pipeline and an ML branch. We utilize the ML branch to label which appliances are present in the datasets with missing sub-meter data. The data processing pipeline consists of eight data processing steps indicated by blue and red colors in Fig. 4, namely: 1. Uniform data format, 2. Load profiles and consumption data, 3. Metadata integration, 4. Data storage and management 5. Relational database to RDF mapping language (R2RML) Mapping, 6. Ontological mapping and RDF generation, 7. Data linking and semantic enrichment 8. Storage in a graph database.

The data contributions proposed in this paper are provided as results of steps 1, 7, and 8, marked with red. Specifically, in step 1, we provide the used datasets in a uniform format stored as pickle files. As a result of step 7, we provide the generated triples that form the KG as a Turtle file, allowing for the expansion of the KG by adding new data properties or incorporating additional datasets as they become available. In step 8, we deploy the KG in a graph database, providing a fully operational SPARQL endpoint (https://sparqlelec.ijs.si/) for our KG, allowing for detailed queries. Additionally, we provide a LodView (https://elkg.ijs.si/) deployment, which visualizes the RDF data and allows users, even those unfamiliar with SPARQL, to explore the KG interactively.

The three ML-related steps, aim at predicting which devices may be present in households that only feature aggregated data. The predicted devices are then inserted in the KG for better modeling off households and their properties. The steps of the ML are colored in purple and consist of (a) Training data generation, (b) Model training, and (c) Predicting appliances. The following subsections elaborate on each of the steps.

#### KG generation pipeline

The KG generation pipeline structures electricity usage data into a queryable graph. It starts by standardizing data to a uniform format for integration. Load profiles and consumption metrics are calculated to analyze electricity consumption. Metadata is added for socio-economic and geographical contexts. The data is then stored in a PostgreSQL database and transformed into RDF triples using R2RML and Ontopic Studio, based on established ontologies. This RDF data is linked to external databases like Wikidata (https://www.wikidata.org/) and DBpedia (https://www.dbpedia.org/) to enhance its value. Finally, the data is stored in a Blazegraph graph database, enabling access and querying through a SPARQL endpoint. A SPARQL endpoint provides an interface for querying the database. This endpoint allows users to retrieve specific information and explore relationships within the data using the SPARQL query language. All these steps and their implementation details are described in the paragraphs bellow. The entire KG generation pipeline can be customized via a configuration file (https://github.com/sensorlab/energy-knowledge-graph/blob/main/configs/pipeline_config.py), allowing users to select specific steps to execute for example, $STEPS=[\mbox{''}parse\mbox{''},\mbox{''}loadprofiles\mbox{''},\mbox{''}metadata\mbox{''},\mbox{''}consumption-data\mbox{''},\,\mathrm{....]}$ and designate the datasets to which these steps should be applied. Additionally, it is straightforward to add new datasets to the pipeline, users only need to write a parser script for the new dataset to convert it to the uniform format allowing it to be used by the other pre-processing scripts.

##### Uniform data format

As discussed in the previous section, the existing data-driven studies related to electricity noticed insufficient measured metrics, sample size, time period, and geographical scale^29^. To date, understanding the contents of existing datasets requires significant reading and development work. With providing a unified format, we consider two criteria (1) *fidelity* and (2) *processing simplicity*. We aim to find and transform as many existing household electricity datasets into a uniform data structure and format that can be loaded and visualized with 2–3 lines of code as opposed to needing to develop a parser for each dataset.

To reach our aim, we first analyze existing open source datasets as listed in Table 1, of sufficient quality and sampling frequency that could be easily extended with other datasets generated by regular homes equipped with standard low-frequency smart meters as per European Union and UK technical specifications (https://www.dlms.com/core-specifications/#COSEM). We observe that the datasets originally come in a variety of formats such as CSV files, parquet, xls and others. We also notice that the content of those files varies significantly with some datasets distributed across multiple files per house, others consolidated into one file per house, or even multiple houses recorded in a single file. These discrepancies pose challenges in developing pre-processing pipelines without first standardizing the datasets. During the data standardization phase, we address these issues by creating specific parsers for each dataset to achieve a consistent format across all data as can be seen in Fig. 2. We also clean the datasets by removing duplicate indices and dropping NaN values as per the *validity* criterion. Following this standardization phase, the data is organized into a nested dictionary structure and provided as pickle files for each dataset as per the *processing simplicity* criterion. This dictionary structure, depicted in Fig. 2, is the same for all datasets with a three level structure as illustrated for the first household in the REDD^30^ dataset in Fig. 2. Each first-level dictionary is keyed by the name of the household, leading to a secondary dictionary. This secondary level is composed of keys representing individual appliances and the aggregate consumption of that household, with the corresponding values being the dataframes specific to each appliance containing a datetime index and power consumption in watts. Once the datasets are in the uniform data format they can then be further utilized by our open-source tools to structure and enrich them to create the KG as depicted in Fig. 4. Furthermore it can be utilized as is to study or develop various models such as load/flexibility forecasting, etc., as listed in the applications box in Fig. 1.

##### Load profile and consumption data calculation

**Fig. 5:**
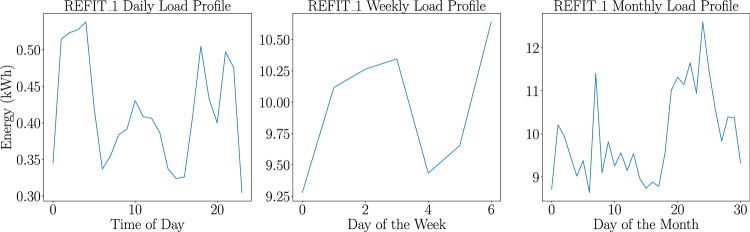
Daily, weekly, and monthly load profiles for household 1 in the REFIT dataset^31^. The daily load profile shows energy consumption across different hours of the day, revealing peak usage times. The weekly profile highlights variations in energy use throughout the week, while the monthly profile captures consumption trends across different days of the month. These profiles provide insights into the household’s energy usage patterns over various time scales.

Load profiles are computed for each household and each appliance on a daily, weekly, and monthly basis by averaging hourly energy consumption in kWh (for daily profiles) and aggregating data on a daily basis (for weekly and monthly profiles), aiding in the understanding of user behavior and consumption patterns. Figure 5 illustrates daily, weekly, and monthly load profiles from household 1 in the REFIT^31^ dataset. The y-axis shows energy used (kWh), while the x-axis represents the time of day (daily), or day of week/month (weekly/monthly). Such information offers insights for distributors and policymakers to aid in reducing peak demand^1^. In this step, we also calculate the electricity consumption data at a household and appliance level. We compute the average daily consumption in kWh and additionally, for appliances, we also determine the average ON/OFF event power consumption in kWh. We utilize this data to calculate the carbon footprint of the household, which can help governments shape policies to reduce the carbon footprints of households with high emissions through subsidies and taxes. Additionally, this data is also useful for the residents of the household, who can alter their behavior in an attempt to reduce their carbon footprint by aligning their consumption with times of peak renewable production, if possible.

##### Metadata integration

Some of the datasets listed in Table 1 contain metadata such as house type, occupancy, what direction they are facing, latitude, longitude, etc. as listed in the upper part of Table 2. We see from the upper part of Table 2 that different households have varying levels of metadata depending on dataset availability for statistics such as occupancy and house size. For instance, in columns two and three, REFIT_1 includes information about occupancy, while HES_1 does not. Location data is also variable: we might have exact household coordinates or just country-level information. For example, in the ECO dataset, we only know that the households are located in Switzerland, resulting in missing data about population density, elevation, city and street details. The metadata collected from all datasets in Table 1, along with higher-level features derived from data analysis (such as load profiles and average daily consumption patterns), is systematically organized in the KG according to the ontology schema shown in Fig. 3. This schema is structured around key classes that represent different aspects of the data. Location-related metadata (such as population density, coordinates, and GDP) is encapsulated in the *Location* class (bottom right of the schema), with associated properties capturing these geographical and demographic characteristics. Meanwhile, consumption-related information extracted from the time series data is represented by the *Device* class (top left), which captures device-specific usage patterns. Finally household specific characteristics (such as number of occupants, start and end date of measurements, house type) are encoded using the *House* class (top right) and its properties.

**Table 2:** Metadata examples for three different households within the KG, showcasing the variability in available properties across datasets.

Properties from datasets	REFIT_1	HES_1	ECO_2
house type	house	house	/
start date	2013-10-09	2018-05-12	2012-06-01
end date	2015-07-10	2018-10-10	2013-01-31
occupancy	2	/	/
facing	/	/	/
num_rental_units	/	/	/
ev_battery_size	/	/	/
house_size	/	/	/
daily household consumption	9.96	64.91	3.87
lat	52.77	45.508	/
lon	−1.2097	−73.561	/
country	United Kingdom	Canada	Switzerland
continent	Europe	North America	Europe
city	Charnwood	Montreal	/
street	Granby Street	Boulevard Saint-Laurent	/
Properties from external sources			
GDP	43192	48962	66661
wages	51594	56083	69383
population density	3197	9045	/
elevation	44	29	/
education level	HIGH	HIGH	MEDIUM
electricity price	0.281	0.279	0.254
gas price	0.065	0.056	0.0617
carbon intensity	268.3	128.46	47.1

The table includes information such as geographical location (continent, country, city, street, latitude, and longitude), socio-economic indicators (GDP, wages, population density, elevation, and education level), energy-related data (electricity and gas prices, carbon intensity), and household-specific attributes (house type, start and end dates, occupancy, and daily household consumption). The table is divided into two sections, where properties from datasets are presented first, followed by properties from external sources, highlighting the direct and indirect sources of data.

When city or country level information are available in the datasets from Table 1, we use them to integrate external data, covering socio-economic metrics such as Gross domestic product (GDP), average income, education attainment levels, and prices of gas and electricity, along with population density as listed in the lower part of Table 2 and Fig. 3 in the *Location* class marked in red. Further exploration into the meaning of the properties can be found in the ontological definition of the entities and properties in the KG (https://elkg.ijs.si/ontology/isFacing). This external metadata can help identify regions with higher CO_2_ footprint and can help policymakers create targeted subsidies for such areas to reduce their carbon footprint which would not be possible otherwise. Moreover, understanding the interplay between income levels and carbon footprints, as evidenced by the finding that lower-income homes have a disproportionately higher energy usage intensity^32^, underscores the importance of leveraging metadata to design equitable and effective transition strategies towards low-carbon heating solutions.

##### Data storage and management

Processed, and enriched with metadata, load profiles, and consumption data, the households are organized within a PostgreSQL database to support efficient data management and retrieval. This database also serves as an interim storage solution for our data before we generate triples using an R2RML mapping as described in the next paragraph. This method of using relational databases as an intermediary step in KG development is a common practice^28^. The data regarding households is stored in a *Household* table, which is linked through foreign keys to a *Location* table containing location-specific metadata and a *Device* table detailing appliance information. This configuration also facilitates the use of PostgresML, enabling the execution of ML operations directly within the database using SQL, leveraging the stored data.

##### Ontological mapping and RDF generation

To generate the RDF triples used to populate the KG, we need a mapping from the PostgreSQL database tables to RDF triples. For this, we utilize R2RML, a mapping language that maps from relational databases to RDF triples. To generate this mapping we utilize Ontopic Studio an R2RML no code mapping tool, where we map from the PostgreSQL database tables to RDF triples, guided by the SAREF and schema.org ontologies, along with a custom ontology for domain-specific properties like gas and electricity prices. The ontology is visualized in Fig. 3. Taking the household table as an example, we map the *house_size* column to the predicate *schema:floorSize*. This mapping means that each row in the *house_size* column generates a corresponding triple in the KG.

##### Data linking and semantic enrichment

In our research, we aim to significantly enhance the interoperability and value of our KG by linking it with established knowledge bases such as Wikidata and DBpedia, focusing particularly on cities and countries. By linking the cities and countries in our knowledge graph to their WikiData and DBpedia counterparts, it adds even more metadata like population statistics, geographic properties, economic indicators, demographic data and more. This linking significantly enhances the depth and breadth of information available in our knowledge graph, additionally, it provides standardized identifiers and references, facilitating easier integration with other datasets and applications that utilize these widely-recognized knowledge bases. For country linkage, we directly query the SPARQL endpoints using unique country names, enabling us to associate our *schema:Country* entities with their corresponding entities in Wikidata and DBpedia through the *owl:sameAs* predicate. City linkage presents a greater challenge due to the common occurrence of cities sharing names across different locations. To address this, we initiate our process by querying for settlements within a 50-kilometer radius of a household’s coordinates from Wikidata and DBpedia endpoints. We then apply string matching using Python’s FuzzyWuzzy library to find the correct city from the list of settlements. If an exact match is not found, we select the nearest city to the household coordinates for linkage. Finally, we link our *schema:City* entity to the matching entity in WikiData or DBpedia using the owl:sameAs predicate, ensuring accurate and meaningful connections within our KG. The newly generated triples are then inserted into our KG through the SPARQL endpoint.

##### Storage in Graph Database

RDF triples are stored in a Blazegraph database, accessible at our SPARQL endpoint (https://sparqlelec.ijs.si/sparql), which provides robust querying capabilities, data persistence, and enhanced visualization options. The Blazegraph database allows for detailed queries of the data using SPARQL. It also supports the LODView deployment that runs on top of it, allowing easy exploration of the data and visualizing the RDF as HTML. This combination provides a powerful and user-friendly interface for researchers and data analysts to interact with the knowledge graph. LODView offers an intuitive way to navigate through the linked data, presenting the RDF triples in a human-readable format and enabling users to follow connections between entities. Furthermore, Blazegraph’s advanced indexing and query optimization techniques ensure efficient execution of complex SPARQL queries, even on large datasets. Access to a read-only SPARQL endpoint is facilitated via an nginx reverse proxy.

#### Machine learning pipeline

The ML pipeline aims to predict which devices are present in a household in which only aggregated measurements are available. This way, the estimated devices can be inserted in the KG thus enriching it. Therefore, using ML, we solve an appliance ON/OFF classification problem. Such data in the KG may be relevant as it has been shown that detailed appliance level consumption data can decrease overall consumption by up to 15%^15^.

The pipeline starts by creating a training dataset from sub-metered electricity usage datasets. This involves standardizing appliance names, resampling data to a consistent interval, and correcting data anomalies. The dataset is segmented and balanced to simulate various household energy consumption patterns. This process is described in more detail in the next, training data generation paragraph.

The model, an adapted version of InceptionTime^33^, which we selected for its superior classification performance demonstrated across multiple studies for time series classification^34,35^, is trained on this dataset to predict the appliance’s ON/OFF status, employing a multi-label classification approach. Training involves an ensemble of models to ensure robustness, with adjustments for learning rate and early stopping to optimize performance. The pipeline aims to accurately identify appliances present in the household just from the aggregate consumption. These predictions are then added to the KG via a SPARQL query. The ML pipeline is fully configurable via a configuration file (https://github.com/sensorlab/energy-knowledge-graph/blob/main/configs/model_config.py), where users can specify various parameters, including model hyperparameters (learning rate, batch size, number of epochs), dataset generation parameters (window size, number of windows, sampling rate), and testing parameters.

##### Training data generation

**Fig. 6:**
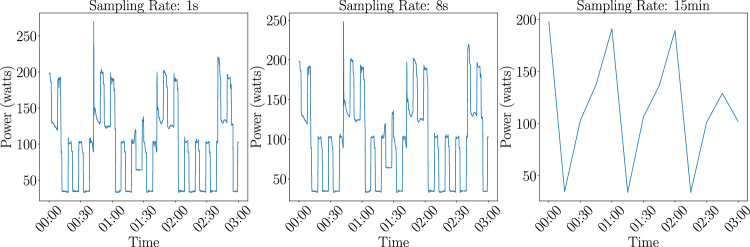
Impact of sampling rate on the Visibility of appliance usage events over time. It can be seen that the loss of detail between 1 s and 8 s is minimal and we can still clearly see appliance signatures, while with a 15 min sampling rate the loss of details is significant.

We utilize a comprehensive array of sub-metered datasets with sufficient sampling rates. Datasets with lower sampling rates can not be used for appliance ON/OFF classification as they are not detailed enough to be able to identify individual appliance traces. Figure 6 highlights the loss of fine-grained patterns essential for appliance ON/OFF classification at the 15-minute sampling which is the standard in the field of energetics. This loss of resolution contrasts with the detail preserved at 1 s and 8 s sampling rates, which allows for the distinction of individual appliance activity.We use DEDDIAG^36^, DRED^37^, ECO^38^, ENERTALK^29^, HEART^39^, HES^40^, IAWE^41^, REDD^30^, REFIT^31^, and UKDALE^42^, to train our model. The training data is generated from the uniform data format as can be seen in Fig. 4 in steps 1. and step a) of the ML pipeline The training process begins with data harmonization, where we employ string matching techniques to unify the nomenclature of appliances across these datasets, such as equating ‘fridge’ with ‘refrigerator’. We exclude appliances, such as outlets due to the ambiguity in what is connected to them. All datasets are then resampled to a uniform 8-second interval, a decision informed by the lowest sampling rate present in the REFIT^31^ dataset, while also adhering to the EU technical specifications dictating that smart meters must have a sampling rate of 10 seconds or less (https://www.dlms.com/core-specifications/).

We treat negative power consumption values as anomalies since our training datasets do not include households with photovoltaics, and the negative values are typically very small, such as -0.0001. For appliance on/off classification, we focus exclusively on positive power consumption, as the model predicts appliances based on their power traces.

**Fig. 7:**
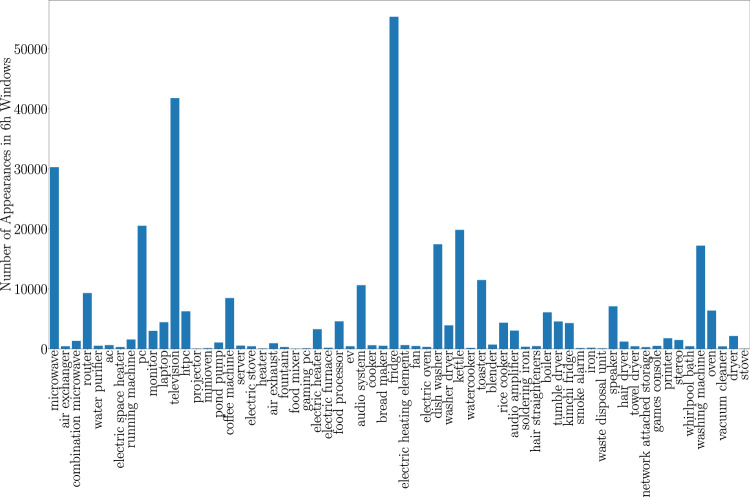
Distribution of appliance representation within data windows. The frequency of each appliance type indicates the number of 6 hour windows in which the appliance is active.

Following this, we segment the data from each appliance for all households into 6-hour windows. With this segmentation we create a set of samples for each appliance from which we generate synthetic households. The 6 hour window size was chosen to capture the full range of potential appliance operation cycles, including longer-running appliances such as washing machines. We explored various window sizes (30 minutes, 1 hour, 3 hours, and 12 hours), finding that window sizes within this range had a negligible impact on model performance. Windows lacking significant appliance activity, containing too many missing values, or exhibiting unrealistic values are discarded. To mitigate potential bias due to certain appliances dominating the dataset with a higher frequency of representative data windows as seen in Fig. 7, We simulate synthetic households for dataset balancing. We sample the number of appliances based on a normal distribution and randomly select the corresponding number of appliances from our entire appliance pool. For each chosen appliance, a random 6-hour window of data from that appliance is selected, and the energy consumption from the selected appliances is aggregated to calculate the total energy consumption measurement. This aggregated data, forming a vector of length 2688 representing household power consumption sampled every 8 s, is then normalized using min-max normalization and fed into our ensemble of models. For dataset construction, we generate 100,000 of these windows, allocating 80,000 for model training and 20,000 for testing.

##### Model training

**Fig. 8:**
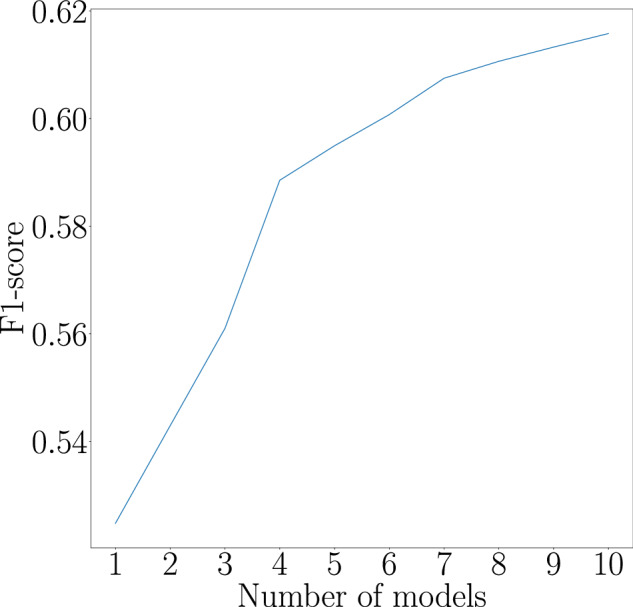
The graph shows the effect of increasing the number of models in an ensemble on the F1-score. As the number of models increases from 1 to 10, the F1-score improves, with the most significant gains observed up to 5 models. Beyond that, performance gains continue but at a diminishing rate.

**Fig. 9:**
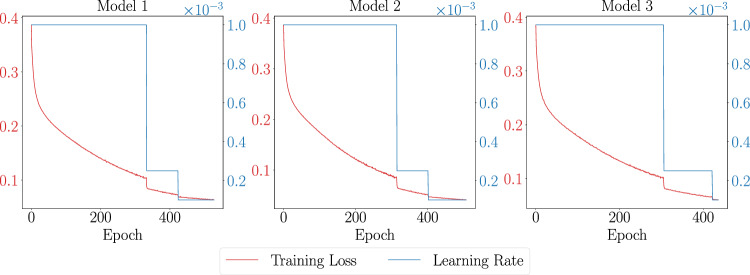
Training loss and learning rate over epochs for 3 randomly chosen models from the ensemble. Each plot shows the relationship between the training loss (red curve) and the learning rate (blue curve) as the models are trained over 500 epochs. The training loss decreases steadily across epochs, indicating that the models are learning and improving over time. The learning rate is adjusted dynamically during training, initially starting high to allow rapid learning, and then gradually decreasing to fine-tune the models and stabilize the loss.

To develop an appliance classifier using the sub-metered datasets from Table 1 and the training data generation from step a), we selected the InceptionTime^33^ architecture, a state-of-the-art time-series processing neural network proven to have excellent performance on multi-class time series classification which we adapted for multi-label classification. This adaptation involves modifying the loss function to Binary Cross-Entropy and substituting the final layer’s activation function with a sigmoid to accommodate for multi-label classification. The architecture consists of an ensemble of Inception classifiers and the ensemble size as well as the input size can be tuned to accommodate different tasks. During the model training step, we used an ensemble of 10 models with an input size of 2688. We opted for an ensemble of 10 models because it enhances the F1-score by over 10% compared to using a single model. We did not increase the ensemble size beyond this as the performance gains show diminishing returns; the improvement in the F1-score between using 7 and 10 models is less than 1% at a significant computational cost. This trend is illustrated in Fig. 8, which plots the F1-score on the y-axis against the number of models in the ensemble on the x-axis. The input represents the aggregate consumption of the household in a 6-hour window with a sampling rate of 8 s, and the training target is a binary vector of length 64 that represents the ON/OFF status of appliances in the given time window. The models are trained for up to 1200 epochs, incorporating a learning rate that diminishes when a plateau in performance is detected. Additionally, we implement an early stopping mechanism that halts training upon the models’ convergence, which typically occurs around the 450-epoch mark. This training strategy is visualized in Fig. 9, which displays the progression of training loss and the adjustments in learning rate for three models from our ensemble. A notable observation from the figure is the initial learning rate reduction, which occurs approximately at the 320-epoch juncture, illustrating the adaptive nature of our training methodology to optimize model performance.With this training methodology, our model achieved an average F1-score of 0.62 on the test set with 64 appliances across three runs with different seeds.

##### Predicting appliances

**Fig. 10:**
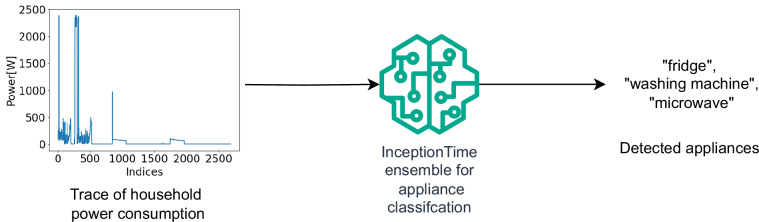
This figure shows a sample training data instance and the output from our ensemble of InceptionTime models. The trace of a running washing machine is visible between indices 0 and 500. Repeated traces of a fridge are observed around indices ∼1000 and ∼2000, spanning the entire sample. While the spike at index ∼900 represents a microwave.

To apply our trained model to an unlabeled dataset to detect the presence of appliances, we follow these steps: First, we standardize an unlabeled, previously unseen dataset, with a sampling rate of at least 8 seconds. If the dataset has a higher sampling rate we down-sample it to 8 s. Next, we divide the dataset into segments spanning 6 hours each, discarding any segment without active appliance readings or with excessive missing data. Subsequently, we normalize the data within each segment using a min-max normalization. Finally, these prepared segments are fed into our model. We then average the output probabilities over all the windows. To determine the presence of appliances, we apply thresholding. In this process, a threshold value (in our case, 0.3), chosen based on experiments conducted on labeled data is selected. Any appliance with an average probability exceeding this threshold is considered present within the household. As depicted in Fig. 10, a sample consumption window and the corresponding model prediction illustrate this process. The figure shows an example where the trace of a washing machine’s operation is visible between indices 0 and 500, while repeated patterns corresponding to a fridge appear around indices 1000 and 2000, covering the entire sample. Additionally, a spike around index 900 indicates the use of a microwave.

### Comparison to SotA

We compared our model to the current state-of-the-art model for multi-label appliance classification^43^. Both models were applied to our dataset and trained using identical parameters to ensure a fair comparison. The SotA model achieved an F1-score of 0.42, which significantly under performed compared to our proposed ensemble of 10 InceptionTime models, which achieved an average F1-score of 0.62 with a standard deviation of 0.04 across three runs with different seeds. The average F1-score achieved by a single model in the ensembles was 0.52 with a standard deviation of 0.04 across the 30 models, indicating consistent performance with some variation. As shown in Table 3, our ensemble models consistently outperform the SotA baseline across all metrics, with Ensemble 2 achieving the highest performance (F1-score: 0.67, precision: 0.78, recall: 0.61).

**Table 3:** Performance comparison between our ensemble models and the state-of-the-art (SotA) model^43^ across F1-score, precision, and recall metrics.

	Ensemble 1	Ensemble 2	Ensemble 3	SotA
F1-score	0.58	0.67	0.60	0.42
Precision	0.66	0.78	0.68	0.45
Recall	0.55	0.61	0.57	0.43

**Fig. 11:**
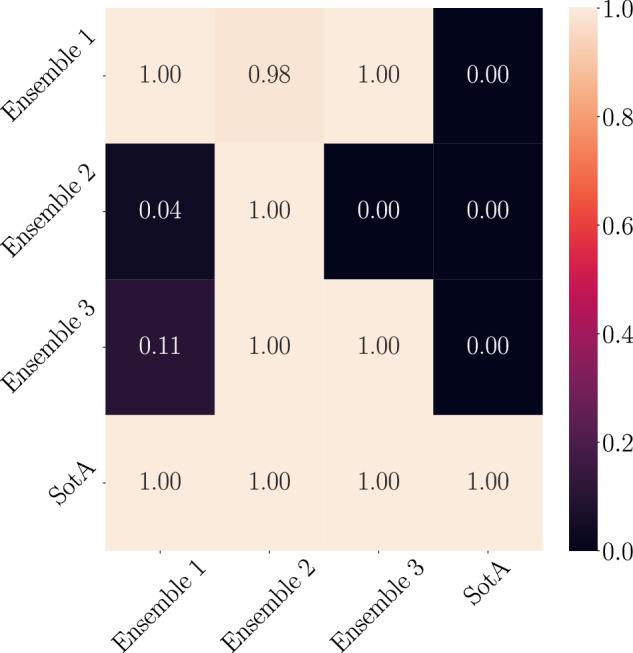
Comparison of 3 runs of our ensemble on different seeds against the SotA model^43^ using Almost Stochastic Order (ASO) with a confidence level of *α* = 0.05. Heat map should be read as ASO (row,column), where each box represents the (*ε*_min_) score of the pair. The lower the score the better.

We also compared the models using a statistical significance test, specifically, we tested with Almost Stochastic Order (ASO)^44^. ASO is a statistical method used to compare the distributions of two random variables to determine if one distribution tends to yield higher values than the other, with allowance for minor deviations. This method involves trimming both distributions to improve the agreement to stochastic order, thereby quantifying the extent to which one distribution is stochastically dominant over the other. Using ASO with a confidence level *α* = 0.05, we found the score distribution of our proposed model based on three random seeds to be stochastically dominant over the SotA model (ε_min_ = 0) as can be seen in Fig. 11. The figure should be read as ASO(row, column). It can be seen from the second row, first and third column in the heatmap that from the three InceptionTime models the 2nd run performs the best, outperforming the other InceptionTime models with (ε_min_ = 0.04) and (ε_min_ = 0) respectively. The lower ε_min_ score the better.

## Data Records

Table 4 showcases the availability of features across different data formats. For instance, raw data includes the raw consumption data and some metadata, but it is stored in various formats across different datasets. In contrast, the proposed uniform data format is presented in a uniform format and contains the raw consumption data, though it does not include metadata. The proposed KG format is also in a uniform format and contains both metadata and enriched metadata from external sources; however, it does not include raw consumption data. Instead, it offers features extracted from the consumption data, such as average daily usage and load profiles. A more in depth analysis of all the data is available in the following subsections. The uniform data and metadata needed for running the pipeline is available to download from our Figshare repository^45^. We provide a full uniform data dump and a small sample to test. The triples constituting the KG are also available to download from this repository.

**Table 4:** Comparison of features provided by Raw Data, Uniform Data Format, and Knowledge Graph.

	Raw Data	Uniform Data Format	Knowledge Graph
Raw consumption data (from datasets in Table 1)	✓	✓	X
Metadata (from datasets in Table 1)	✓	X	✓
Uniform format	X	✓	✓
Enriched metadata (from external sources)	X	X	✓
Extracted features from consumption data	X	X	✓

More details about the metadata are available in the “Metdata Integration” section.

### Raw data dump

The raw datasets utilized in our study, are available for download as tar.gz compressed files in our Github repository (https://sensorlab.ijs.si/archive/energy-knowledge-graph/). To accommodate varying computational resources, we provide two versions of the data dump: a complete and comprehensive version that includes all raw datasets, which is approximately 91.2 GB when compressed with gzip, and individually compressed raw datasets, allowing users the flexibility to download any combination of datasets according to their needs. The raw metadata for all the datasets is available alongside the individual datasets.

### Uniform data format

**Fig. 12:**
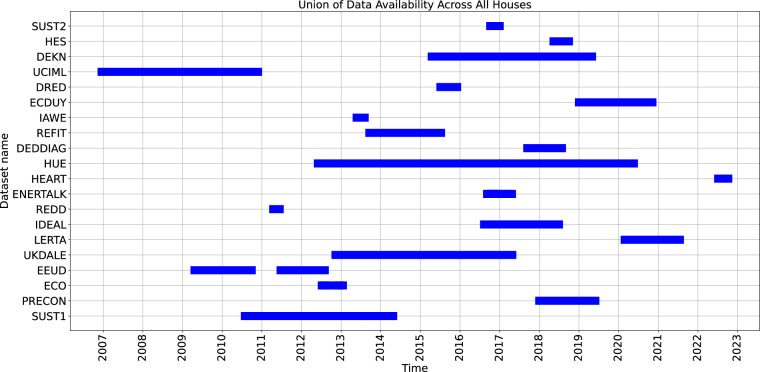
Data availability of power consumption measurements across all datasets. This figure illustrates the periods during which power consumption measurements are available for each dataset. The datasets are listed on the y-axis, and the x-axis represents the time in years from 2007 to 2023. Each blue bar indicates the duration of data collection for the corresponding dataset, showing the union of data availability across all houses within each dataset.

The proposed uniform dataset derived from the datasets summarized in Table 1 encompasses power consumption measurements from 111,424 households across 20 datasets. Figure 12 depicts the breakdown by dataset of when it was recorded and for how long. It can be seen that the data spans from 2007 to 2022. For example, we can see that datasets such as UKDALE^42^ and HUE^46^ span multiple years while some others such as IAWE^41^ and REDD^30^ span only a month. This data is then prepared for further preprocessing and analysis and is also available to download from our Figshare repository^45^.

**Fig. 13:**
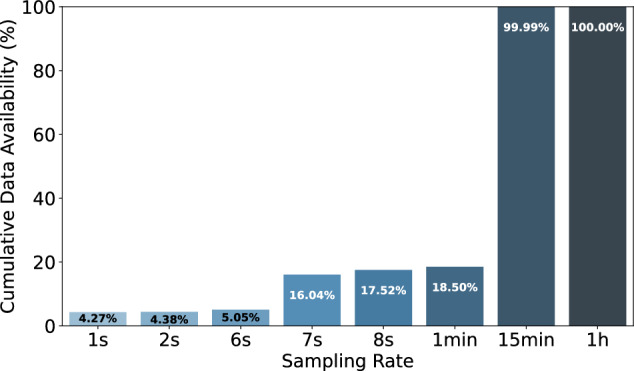
Cumulative percentage of data points available for aggregate power consumption measurements by sampling rate. This figure showcases the distribution of available sampling rates, highlighting that the majority of data points are collected at a 15-minute interval, largely due to the influence of large datasets with less frequent sampling.

**Fig. 14:**
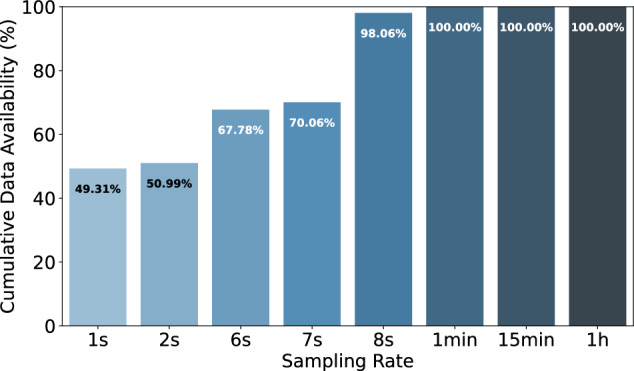
Cumulative percentage of data points available for appliance-specific power consumption measurements by sampling rate. This figure showcases the distribution of available sampling rates for submetered data, with most data points being collected at higher sampling rates, particularly at 8 seconds or higher, which is essential for capturing detailed appliance-level power consumption.

The datasets contain 6,899,283,324 household-level aggregated power consumption measurements and 3,220,934,873 individual appliance power consumption measurements. A more fine-grained breakdown of the average number of measurements per household, appliance, and region is available in Table 5. The table reveals that the majority of appliance-specific (sub-meter) data is collected from European datasets, with approximately 2.6 billion appliance power measurements, compared to 100 million in the Americas and 473 million in Asia. Despite this, the bulk of the households surveyed are in the Americas, primarily due to the ECDUY dataset from Uruguay, which includes 110,953 households. This contributes to a higher total of aggregate household power consumption measurements in the Americas. However, when examining the mean aggregate household power consumption measurements per household, the Americas have significantly fewer data points, approximately 50,839, compared to 3 million in Europe and 2.6 million in Asia. This notable disparity can largely be attributed to the difference in sampling rates; the ECDUY dataset from Uruguay uses a 15-minute sampling interval, whereas most European and Asian datasets feature more frequent sampling rates, often less than one minute. This disparity is also reflected in the duration and scale of data collection; despite having only 350 households spanning 307 years of aggregate household power consumption data, compared to the 164,321 years of aggregate household power consumption data from 111,009 households in the Americas. Yet, Europe has only five times fewer aggregate household power consumption measurements than the Americas, further highlighting the more frequent data sampling in currently available European datasets. This sampling rate and appliance data availability disparity can also clearly be seen in Figs. 13, 14. We can clearly see in Fig. 13 that the majority of aggregate data has a sampling rate of 15 min this is due to the number of households in the ECDUY dataset skewing the distribution. In contrast, Fig. 14 reveals that most appliance data is collected at sampling rates of 8 seconds or higher. The ECDUY dataset does not influence this figure, as it lacks appliance-level data, and sub-metered datasets typically have higher sampling rates to accurately capture the power traces of individual appliances.

**Table 5:** The table compares household and appliance-level power consumption data across Europe, the Americas, and Asia.

	Europe	Americas	Asia
Total household-level aggregated power consumption meas.	1,085,720,074	5,643,637,499	169,925,751
Total individual appliance power consumption meas.	2,647,433,215	99,730,950	473,770,708
Mean household-level aggr. power consumption meas. per household	3,102,057	50,839	2,614,242
Mean individual appliance power consumption meas. per household	7,564,094	898	7,288,780
Total duration of household-level aggr. power consumption in years	307	164,321	42
Total duration of individual appliance consumption in years	623	83	0.25
Total number of appliances	585	139	81
Total number of households	350	111,009	65

Europe leads in individual appliance measurements, while the Americas have the most household-level data, driven by the ECDUY^55^ dataset. Despite fewer households, Europeâ€™s more frequent sampling results in a comparable total measurement volume.

**Fig. 15:**
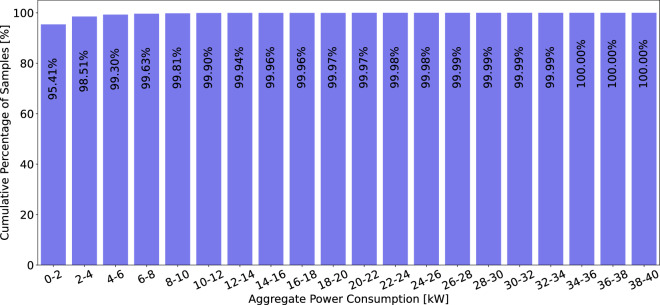
Cumulative percentage distribution of aggregate power consumption data, binned into 20 bins ranging from 0 to 40 kW. This figure illustrates that the majority of the data points are concentrated in the lower power consumption range, particularly within the 0–2 kW bin.

**Fig. 16:**
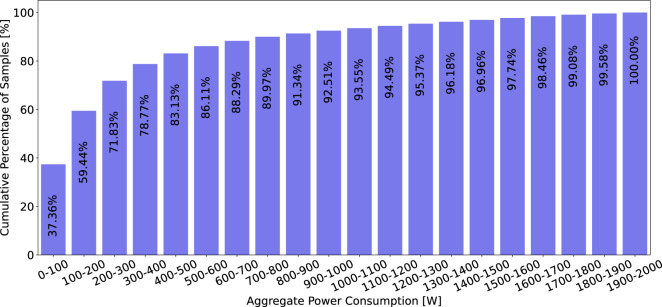
Cumulative percentage distribution of aggregate power consumption data, focusing on the 0–2 kW range from the top figure (Fig. 15). This zoomed-in view highlights the detailed distribution within the most populated bin, where the majority of the data is concentrated.

Further insights can be gleaned by examining the distribution of aggregate power consumption in Fig. 15, we observe that the majority (95.41%) of the measurements fall between 0–2 kW. A closer look at the distribution of measurements under 2 kW in Fig. 16 reveals that nearly 90% of the data points are below 800 watts. This prevalence of low measurements is primarily due to the fact that power-hungry appliances, such as washing machines, dryers, and induction stoves, operate for relatively short periods, while the baseline consumption of less energy-intensive appliances such as refrigerators, televisions and lights dominates the majority of the time.

**Fig. 17:**
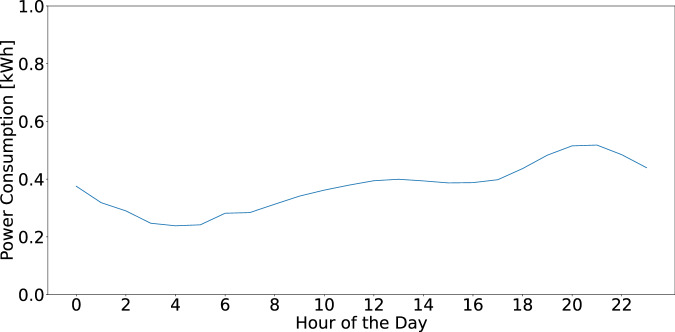
Mean daily load profile of all households across all datasets. This figure illustrates the typical daily pattern of power consumption, with the lowest consumption occurring in the early morning hours, likely when most occupants are asleep. Consumption gradually increases throughout the day, reaching a peak in the evening hours as residents return home before gradually declining again late in the evening.

Finally, if we calculate the load profiles for all households in the datasets, we find that the lowest consumption occurs in the early morning hours when most people are asleep, gradually increasing throughout the day and peaking in the evening when the majority of residents are home and awake. This daily pattern is visualized in Fig. 17.

#### REDD dataset

**Fig. 18:**
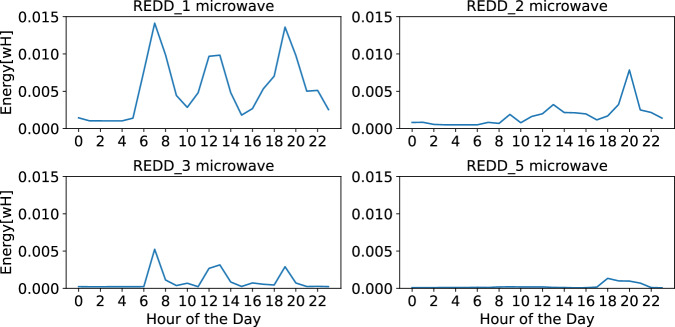
Load profiles of microwave usage in four households from the REDD dataset. Household REDD_1 shows the highest and most consistent microwave usage throughout the day, with peaks suggesting usage for breakfast, lunch, and dinner. REDD_3 follows a similar pattern but with lower intensity. REDD_2 has morning and evening peaks that are shifted by about two hours compared to REDD_1 and REDD_3, with overall lower energy consumption. In contrast, REDD_5 exhibits minimal microwave usage, primarily in the evening, indicating infrequent use.

The REDD dataset provides sub-metered data for various appliances, allowing us to infer certain behavioral patterns of the occupants. In Fig. 18, we observe the load profiles of microwaves for the four households that include this appliance. Household REDD_1 shows the highest microwave usage, with consistent activity throughout the day, suggesting that the occupants are likely home during the day. There are three distinct usage peaks in REDD_1: one in the morning around 8:00, one around midday, and another in the evening around 19:00. These peaks suggest that the microwave is used to prepare breakfast, lunch, and dinner. Household REDD_3 has a similar pattern to REDD_1, with peaks at corresponding times, but the usage is less intense. This could indicate shorter microwave usage durations or a microwave with lower power consumption. Household REDD_2 also shows a similar load profile but with morning and evening peaks occurring approximately two hours later than those in REDD_1 and REDD_3. The overall energy consumption in REDD_2 is more akin to REDD_3, with nearly three times less energy usage compared to REDD_1. In contrast, household REDD_5 exhibits minimal microwave usage, predominantly in the evening, suggesting that the occupants use the microwave infrequently and mainly for evening meals.

### Knowledge graph

Our KG is composed of 791,813 nodes (i.e. instances of subjects or predicates) interconnected through 1,577,483 instances of predicates totaling 1,577,483 triples. It is generated from 6 unique subject/object concepts and 38 unique predicates that form the vocabularies summarized in Table 6. It encompasses 111,423 instances classified under *schema:House* class representing individual households, and details such as average daily consumption and load profiles on 113,359 unique sub-meters and meters that measure per appliance or aggregated consumption. The households span 54 unique locations across 14 countries and 12 cities, underscoring the KG’s comprehensive global perspective and its potential utility for diverse regional energy analyses and applications. Each location instance contains various metadata pertaining to the location, such as GDP, average wage, electricity prices, etc., and is also linked to external knowledge bases such as WikiData and DBpedia further expanding the available location-specific properties. Some of the available metadata can be seen in Table 2 Access to this KG is facilitated through a SPARQL endpoint (https://sparqlelec.ijs.si/sparql), and for those interested in a more granular exploration and customization/expansion of the KG, we offer a downloadable dump of the RDF triples constituting the graph at our Figshare repository^45^. We also provide a visual representation of our RDF data with LodView (https://elkg.ijs.si/resource/public-households/REFIT_1), where we display load profile plots and other properties of the KG.

**Table 6:** Summary of the KG data used in this work, detailing the structure and scope of the graph.

KG entites	Quantity
Total triples	1,577,483
Total instances of predicates	1,577,483
Total unique predicates	38
Total instances of nodes/ subject or object	791,813
Total unique concepts/ subject or object	6
Instances of *schema:House*	111,423
Instances of *saref:Device* (sub-meters and meters)	113,359
Instances of *saref:Device* corresponding to appliances	1936
Instances of *schema:Continent*	4
Instances of *schema:Country*	14
Instances of DBpedia countries	14
Instances of Wikidata countries	14
Instances of *schema:City*	12
Instances of DBpedia cities	12
Instances of Wikidata cities	12

The table provides a breakdown of key entities and their quantities within the KG.

## Technical Validation

### Uniform data format validation

In the process of creating the uniform data format, we analyze open-source datasets, as referenced in Table 1. During this analysis, we eliminate any instances of missing data and convert timestamps to datetime objects, which are then used as indices in pandas. The processed data is organized into a nested dictionary format and preserved as a pickle file. It’s important to note that we do not employ interpolation or other methods to fill in missing data, ensuring the integrity and quality of the original dataset remain intact.

### Metadata validation

For household-specific metadata, we directly use the information provided in the datasets without modification. In terms of spatial data, we utilize the available household coordinates or, in their absence, the country information. It’s important to note that when only country information is available, without precise household locations, we are unable to enrich the dataset with specific details such as elevation and population density. This spatial data then serves as a basis to link with external sources for further enrichment with socio-economic factors as described in more detail in the methodology section.

### Mapping validation

Using Ontopic Studio^47^, we create R2RML mappings to facilitate the transformation of data from a PostgreSQL database into RDF triples. These mappings strictly follow the standards set by schema.org, SAREF, and our custom ontology. The mapping process guarantees error-free conversion of database data into RDF triples, thus preserving the data’s integrity and ensuring its accurate representation In RDF format.

### Linking validation

We use a custom Python script to link our KG to WikiData and DBPedia. We link our *schema:City* and *schema:Country* entities using the *owl:sameAs* predicate to their corresponding counterparts in WikiData and DBPedia. For countries, direct queries to the SPARQL endpoints using country names are sufficient, given the uniqueness of country names. City connections, however, require a two-step process: initially, we query for all settlements within a 50 km radius of a household’s coordinates. Subsequently, we employ string matching to identify the accurate city. In cases where a direct match proves elusive, we default to the nearest settlement to the household’s coordinates, ensuring the most accurate linkage possible.

### Predicted appliances validation

We employ a model to categorize the unlabeled datasets, achieving an average F1-score of 0.62 across 64 classes. Due to this, the identification of appliances may not always be accurate. Consequently, each household is assigned a property, *voc:isSubmetered*, which indicates the reliability of the appliance data. If true, it signifies that the dataset includes sub-meter data, confirming the appliances present as ground truth. However, if false, it implies that the appliances were identified using our model, and their accuracy may be uncertain.

## Usage Notes

### Preprocessing and training pipeline

We provide Python scripts for our preprocessing and model training pipeline in our github repository (https://github.com/sensorlab/energy-knowledge-graph) with detailed instructions on how to use them in the form of README files.

### Knowledge graph

Households and their metadata can be accessed via the provided SPARQL endpoint (https://sparqlelec.ijs.si/sparql), here we provide an example SPARQL query in Listing 1, there are more SPARQL query examples available in our github repository (https://github.com/sensorlab/energy-knowledge-graph).
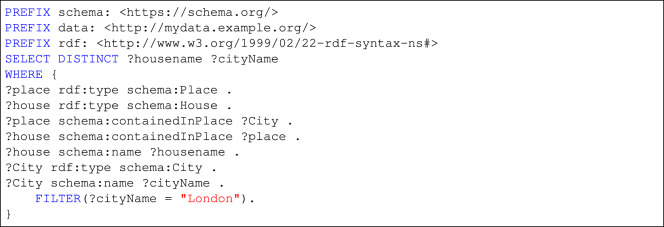


**Listing 1**. SPARQL query to retrieve all households located in London.

## Data Availability

Our source code to preprocess the data and generate the KG is available at our Github (https://github.com/sensorlab/energy-knowledge-graph) repository. Our code is provided under the BSD 3-Clause License.
